# Biportal endoscopic posterior lumbar decompression and vertebroplasty for extremely elderly patients affected by lower lumbar delayed vertebral collapse with lumbosacral radiculopathy

**DOI:** 10.1186/s13018-021-02532-0

**Published:** 2021-06-14

**Authors:** Min-Seok Kang, Dong-Hwa Heo, Hoon-Jae Chung, Ki-Han You, Hyong-Nyun Kim, Jun-Young Choi, Hyun-Jin Park

**Affiliations:** 1 Department of Orthopedic Surgery, Bumin Hospital, Republic of, Seoul, Korea; 2 Department of Neurosurgery, Bumin Hospital, Seoul, Republic of Korea; 3 Department of Orthopedic Surgery, Spine Center, Kangnam Sacred Heart Hospital, Hallym University College of Medicine, 1, Singil-ro, Yeongdeungpo-gu, Seoul, Republic of Korea 07441

**Keywords:** Delayed vertebral collapse, Osteoporotic vertebral compression fracture, Lumbosacral radiculopathy, Extremely elderly patients, Biportal endoscopic posterior lumbar decompression, Vertebroplasty

## Abstract

**Background:**

Lower lumbar osteoporotic vertebral compression fracture in extremely elderly patients can often lead to lumbosacral radiculopathy (LSR) due to delayed vertebral collapse (DVC). Surgical intervention requires posterior instrumented lumbar fusion as well as vertebral augmentation or anterior column reconstruction depending on the cleft formation and intravertebral instability. However, it is necessary to decide on surgery in consideration of the patient’s frail status, surgical invasiveness, and rehabilitation. In the lower lumbar DVC without intravertebral instability, biportal endoscopic posterior lumbar decompression and vertebroplasty (BEPLD + VP) can be simultaneously attempted. This study aimed to assess the clinical outcomes of BEPLD + VP for the treatment of DVC-related LSR.

**Methods:**

This retrospective case series enrolled 18 consecutive extremely elderly (aged ≥ 75-year-old) patients (6 men and 12 women) who had lower lumbar (at or below L3) DVC-related LSR. Patients who require anterior column reconstruction, such as cleft formation accompanied by intravertebral instability and patients who have not been followed for more than 6 months, were excluded from this study. All patients underwent BEPLD + VP under epidural anesthesia. Clinical results were evaluated by the visual analog scale (VAS) score and the modified Japanese Orthopedic Association (mJOA) scores.

**Results:**

Most of the patients had DVC affecting level L4, with the deformation being a flat type or concave type rather than a wedge type. The VAS score (back and leg) significantly decreased from 7.78 ± 1.17 and 6.89 ± 1.13 preoperatively to 2.94 ± 0.64 and 2.67 ± 1.08 within 2 postoperative days (*p <* 0.001). The mJOA score significantly improved from 4.72 ± 1.27 preoperatively to 8.17 ± 1.15 in the final follow-up (p *<* 0.001). The mean recovery rate (RR) in the last follow-up was 56.07% ± 9.98. Incidental durotomy was reported in two patients and epidural hematomas in another two patients; however, all patients improved with conservative treatment, and no re-operation was required.

**Conclusions:**

BELPD + VP was a type of salvage therapy that reduces surgical morbidity, requires major spine surgery under general anesthesia and provides good clinical outcomes in extremely elderly patients with DVC-related LSR.

## Introduction

Osteoporotic vertebral compression fractures (OVCFs), the most common fractures occurring in elderly people, can be managed conservatively and are known to have a benign natural course. However, studies have shown that approximately 30% of all OVCFs presents with progressive collapse, 13% accompanied with nonunion, and 3% develops delayed vertebral collapse (DVC) with neurologic deficits [1, 2]. Moreover, osteoporosis may impair bone healing of the vertebral fracture, causing a collapse in the vertebral body and intervertebral disk height, aggravating segmental kyphosis, and consequently resulting in spinal segmental instability [3–5].

For patients who have developed neurologic symptoms, surgical treatment is a necessity to allow them to return to a daily routine. In patients at an advanced age, lower lumbar DVC affecting at or below L3 causes lumbosacral radiculopathy (LSR) [2]. Furthermore, DVC affecting the thoracolumbar junction is accompanied by compressive myelopathy and paralysis, and neurological symptoms may be improved by short-segment posterior lumbar arthrodesis surgery. The problem arises when fragile bone quality, sarcopenia, global spinal imbalance, and medical frailty together pose a challenge and an ethical dilemma toward the treatment of lower lumbar DVC-related LSR in extremely elderly patients [2, 6].

The authors postulated that minimally invasive neural decompression and anterior column stabilization would reduce the need for instrumented spinal fusion surgery under general anesthesia and could achieve acceptable clinical outcomes for extremely elderly patients having lower lumbar DVC-related LSR. This study aimed to examine the records of minimally invasive interventions consisting of biportal endoscopic posterior lumbar decompression combined with vertebroplasty (BEPLD + VP) for the treatment of lower lumbar DVC-related LSR in extremely elderly patients and to evaluate related clinical outcomes.

## Methods and materials

### Ethical statements

This study was approved by the Institutional Review Board of our Institution and performed in accordance with the ethical standards laid down in the 1964 Declaration of Helsinki and its later amendments. Informed consent was obtained from all patients.

### Patients

Although there is no consensus on the concept of old age, the World Health Organization (WHO) defines the elderly as 60–74 years of age and extremely elderly as 75–89 years of age [7]. Based on this definition, we retrospectively reviewed the medical records of 52 extremely elderly patients aged > 75 years who underwent surgeries for DVC from 2017 to 2019 in a single institution. The inclusion criteria were as follows: aged ≥ 75 years; vertebral height loss of ≥ 50%; magnetic resonance imaging (MRI) showing the morphology of DVC, such as vertebra plana or concave vertebra, with or without intravertebral cleft; and clinically unilateral, bilateral, and/or intermittent neurogenic claudication. Patients who had segmental kyphotic changes, intravertebral instability [2], had undergone lumbar decompressive laminectomy and/or arthrodesis or had been diagnosed with an old compression fracture at/below L3, pyogenic spondylitis, inflammatory spondylitis, spinal tumor, or systemic infection were excluded from the study.

Among these patients, we enrolled 18 (6 men and 12 women) patients whose vertebral (L3 and below) level was affected and had DVC-related LSR. Based on the electronic medical records, comorbidities included hypertension (n = 9), type 2 diabetes (n = 8), ischemic heart disease (n = 4), Cushing’s syndrome (n = 2), and Alzheimer’s disease (n = 2). The initial neurologic status according to the American Spinal Injury Association (ASIA) impairment scale was at grade C in 3 patients and grade D in 15 patients (Table 1) [8].

**Table 1:** Patients’ demographic data

Case no.	Sex	Age (years)	^*^AIS	BMD	Osteoporotic vertebral collapse		Combined spondylotic conditions		Surgical treatment
				Lowest T score	Level	Type	Lumbar central stenosis	Lumbar foraminal stenosis	
1	F	77	D	−3.2	L4	Concave		L4-5: Left	PVP of L4 with BE-UFD of Lt L4-5
2	F	83	D	−2.6	L4	Concave	L3-4: Both		PVP of L4 with BE-ULBD of Lt L3-4
3	F	86	D	−3.4	L4	Concave	L3-4: Both	L3-4: Left	PVP of L4 and BE-ULBD of Rt L34 (CL approach)
4	F	80	D	−3.1	L3	Concave		L2-3: Left	PVP of L3 with BE-UFD of Lt L23
5	F	90	C	−4.1	L4	Concave	L3-4: Both	L3-4: Right	PVP of L4 and BE-ULBD of Lt L3-4 (CL approach)
6	F	92	C	−4.5	L5	Concave	L5-S1: Right		PVP of L5 with BE-ULBD of Lt L5-S1
7	F	75	D	−3.7	L4	Flat	L4-5: Left		PVP of L4 with BE-ULBD of Lt L4-5
8	F	75	C	−2.5	L4	Flat	L3-4: Both	L4-5: Right	PVP of L4 with BE-ULBD of Lt L3-4 and L4-5 (CL approach)
9	M	75	D	−2.7	L5	Concave	L4-5: Left		PVP of L5 with BE-ULBD of Lt L4-5
10	F	81	D	−5.1	L4	Flat	L4-5: Both, L3-4: Both		PVP of L4 with BE-ULBD of Lt L4-5
11	M	88	C	−2.5	L4	Concave	L3-4: Both	L3-4: Right	PVP of L4 with BE-ULBD of Lt L34 (CL approach)
12	F	79	D	−2.7	L3	Flat		L2-3: Left	PVP of L3 with BE-UFD of Lt L2-3
13	M	85	C	−3.3	L4	Flat	L3-4: Both		PVP of L4 with BE-ULBD of Lt L3-4
14	M	78	D	−4.3	L3	Flat	L2-3: Right		PVP of L3 with BE-ULBD of Rt L2-3
15	M	75	D	−2.9	L4	Flat	L4-5: Left	L4-5: Left	PVP of L4 with BE-ULBD of Rt L4-5 (CL approach)
16	F	75	C	−3.1	L4	Flat	L3-4: Left, L4-5: Right		PVP of L4 with BE-ULBD of Lt L3-4 and L4-5 (CL approach)
17	M	77	D	−3.6	L5	Concave		L5-S1: Right	PVP of L5 with BE-UFD of Rt L5-S1
18	F	75	D	−5.1	L4	Flat	L4-5: Left		PVP of L4 with BE-ULBD of Lt L4-5

^*^AIS, ASIA Impairment Scale; *BMD*, bone mineral density; *F*, female; *M*, male; *PVP*, percutaneous vertebroplasty; *BE-ULBD*, biportal endoscopic unilateral laminectomy bilateral decompression; *BE-UFD*, biportal endoscopic unilateral foraminal decompression; *Lt*, left; *Rt*, right; *CL*, contralateral

### Radiological assessment

All patients were diagnosed with DVC using MRI. In this study, MR images were evaluated for changes in bone marrow signal intensity that would be consistent with persistence of bone remodeling at the levels of the vertebral compression fractures, either as simple bone marrow edema with low signal intensity on T1-weighted images, high signal intensity on T2-weighted images, and normal signal intensity on contrast-enhanced T1-weighted images, or as an intravertebral cleft with well-demarcated linear or ellipsoid areas with signal intensity prolongation on T2-weighted images when fluid-filled, or signal intensity void when gas-filled .9 Based on these evaluations, each DVC was classified into one of three types of vertebral collapse.2, 10--12 In type 1 (wedge-type collapse), the ratio of the anterior height of the vertebral body to its posterior height is < 60%. A type 2 collapse (flat-type fracture) encompasses vertebra plana-like fracture with uniform compression. Type 3 (concave collapse or H-shaped fracture) comprises anterior spur formation or sclerotic change [2]. Spinal stenosis was also evaluated and anatomically classified as either central, lateral recess, foraminal, or extraforaminal.

### Surgeries

**Fig. 1:**
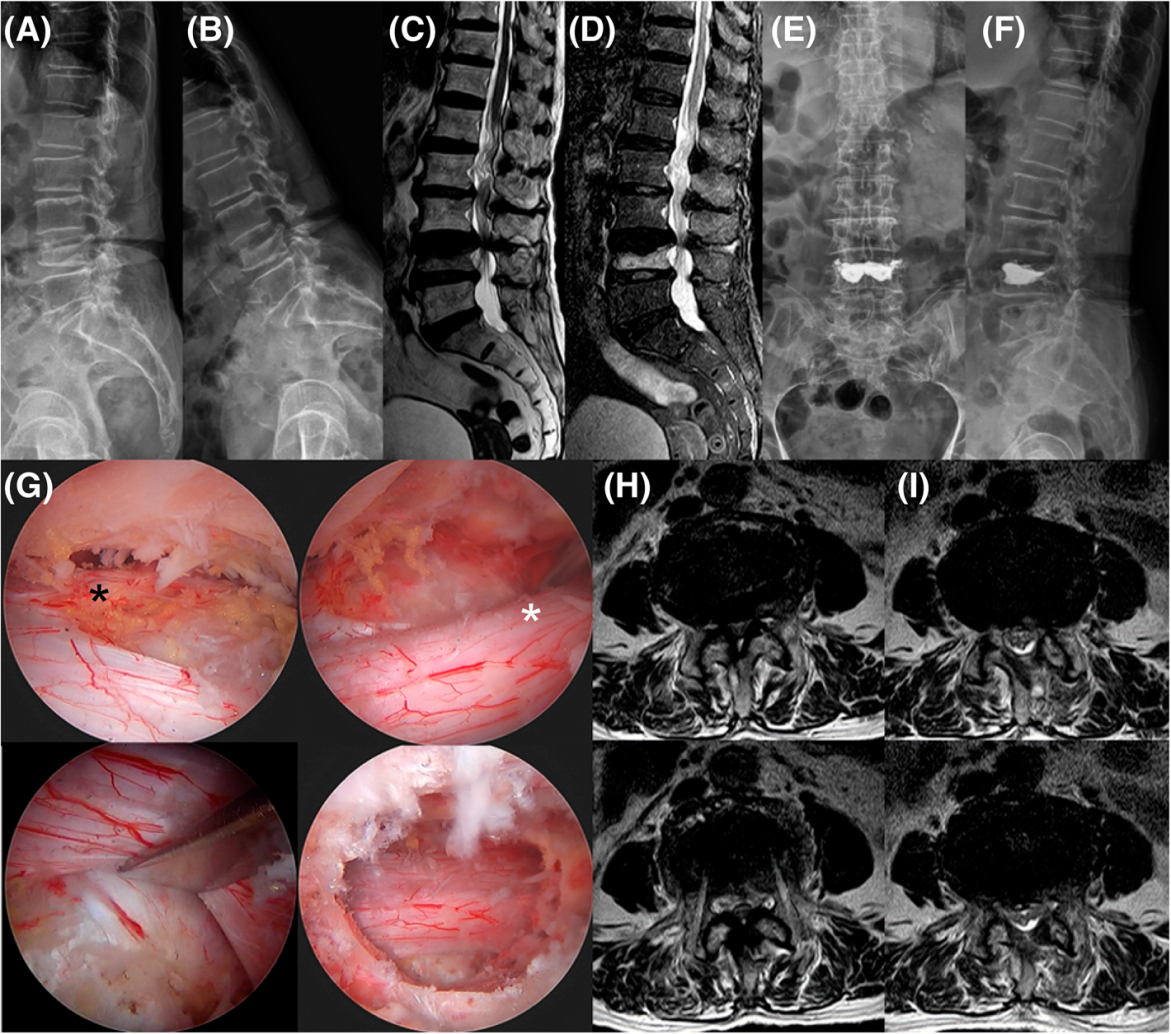
Representative clinical example where surgeries were performed using a uniform technique. A 90-year-old female patient was diagnosed with L4 osteoporotic vertebral collapse (concave-type), severe central canal and right foraminal stenosis at L3-L4, and L3 spondylolisthesis with instability. (**A**) Plain lateral radiographic image. (**B**) Dynamic flexion radiographic image. (**C**) Magnetic resonance T2-weighted sagittal image. (**D**) Magnetic resonance T1-weighted fat-suppression sagittal image. (**E**, **F**) The patient underwent percutaneous balloon kyphoplasty for L4 osteoporotic vertebral collapse but continued to experience severe radicular pain and its related gait disturbance. (**G**) Finally, she underwent unilateral biportal endoscopic decompressive laminectomy. Sufficient neural decompression of the right L3 exiting nerve root (black star), right L4 traversing nerve root (white star), and left L4 traversing nerve root (black cross) are shown by endoscopic visualization. The same can be seen on the preoperative (H) and postoperative (I) magnetic resonance T2-weighted axial images

Although all 18 patients initially received conservative treatment, neurologic symptoms subsequently developed, and vertebral collapse progressed gradually. In 14 patients, BEPLD was performed simultaneously with VP under epidural anesthesia. In the remaining 4, BEPLD was performed when the neurologic symptoms persisted even after a percutaneous VP was performed. In both cases, BEPLD was performed in the manner described by Kim et al. [9–11]. There are two surgical methods of BEPLD, namely, biportal endoscopic unilateral laminectomy bilateral decompression (BE-ULBD) and biportal endoscopic unilateral foraminal decompression (BE-UFD). The surgical method used was determined on the basis of the patient’s symptoms and lesions observed on an MRI scan. Also, VP was performed with a polymethyl methacrylate (PMMA) cement filling through the pedicle of the fractured vertebra. All procedures were performed using a uniform technique (Fig. 1). To avoid bias, two independent assessors, not involved in the surgery, evaluated the postoperative outcomes.

### Clinical outcomes

The operation time, amount of surgical drainage, and volume of the injected PMMA cement were recorded. The dominant dermatome of LSR was assessed and stratified by the morphological features of DVC. All patients completed a visual analog scale (VAS) as an assessment of their pain and a modified Japanese Orthopedic Association (mJOA) scale as an assessment of their neurologic status [12]. VAS score and postoperative complications were follow-up for 12 months (6 weeks and 3, 6, and 12 months postoperatively; mean, 13.4 months; range, 12 to 20 months). Furthermore, the recovery rate (RR) was calculated as follows:

$$ RR\ \left(\%\right)=\frac{\left(\mathrm{final}\ \mathrm{follow}\hbox{-} \mathrm{up}\ \mathrm{mJOA}-\mathrm{preoperative}\ \mathrm{mJOA}\right)}{\left(11-\mathrm{preoperative}\ \mathrm{mJOA}\right)}\times 100 $$

Based on the RR values, the surgical results were classified as good (50–100%), fair (25–49%), unchanged (0–24%), or deteriorated (< 0%) [13, 14].

### Statistical analysis

All values are expressed as mean ± standard deviation (SD). Differences between groups were examined for statistical significance using Student’s t test. A probability value less than 0.05 was considered to represent a significant difference. All statistical analyses were conducted using the SPSS software version 20.0 (IBM Corporation, Armonk, NY, USA).

## Results

**Fig. 2:**
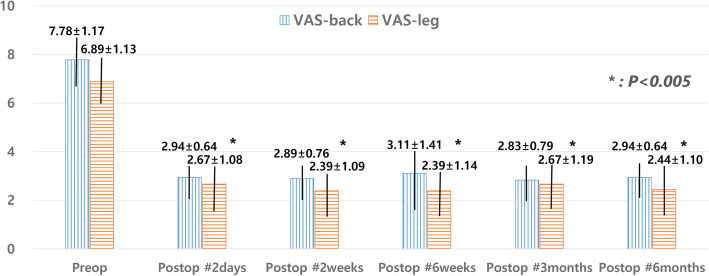
Clinical outcomes based on the visual analog scale (VAS) score. The VAS score (back and leg) significantly decreased from 7.78 ± 1.17 and 6.89 ± 1.13 preoperatively to 2.94 ± 0.64 and 2.67 ± 1.08 within 2 postoperative days (*P* < 0.001). This improvement of the VAS score lasted until the final follow-up, with the score significantly improving to 2.94 ± 0.64 and 2.44 ± 1.10 (*P* < 0.001). Preop, preoperatively; postop, postoperatively

The group included 6 men and 12 women with an average age of 80.04 ± 5.68 years (range, 75–92 years). Clinical symptoms were manifested by unilateral radicular pain in 7 patients, bilateral radicular pain in 11 patients, and neurogenic claudication in all patients. The vertebra level most affected was L4, followed by L3 and L5. Further, the vertebral collapse in most patients was noted as either type 2 (flat-type) or type 3 (concave-type), and no type 1 (wedge-type) classification was observed. Radiographic images from the MRI indicated central canal stenosis in 14 patients, central to subarticular disk herniation in 5 patients, foraminal disk herniation in 2 patients, and foraminal stenosis in 9 patients (Table 1). The mean total operation time was 87.32 ± 10.26 min (range, 80–120 min), the mean amount of surgical drainage was 54.5 ± 20.43 mL (range, 35–120 mL), and the mean amount of PMMA cement was 5.36 ± 1.1 mL (range, 3–8 mL). The mean length of hospital stay was 7.05 ± 3.06 days (range, 4–16 days). No patient required treatment in the intensive care unit. The VAS score (back and leg) significantly decreased from 7.78 ± 1.17 and 6.89 ± 1.13 preoperatively to 2.94 ± 0.64 and 2.67 ± 1.08 within 2 postoperative days (*P* < 0.001). This improvement of the VAS score lasted until the final follow-up, with the score significantly improving to 2.94 ± 0.64 and 2.44 ± 1.10 (*P* < 0.001) (Fig. 2). The mJOA score significantly improved from 4.72 ± 1.27 preoperatively to 8.17 ± 1.15 in the final follow-up (*P* < 0.001). The mean RR in the last follow-up was 56.07 ± 9.98%. Based on the RR values, 15 cases were classified as good and 3 cases as fair; no case was classified as deteriorated.

**Fig. 3:**
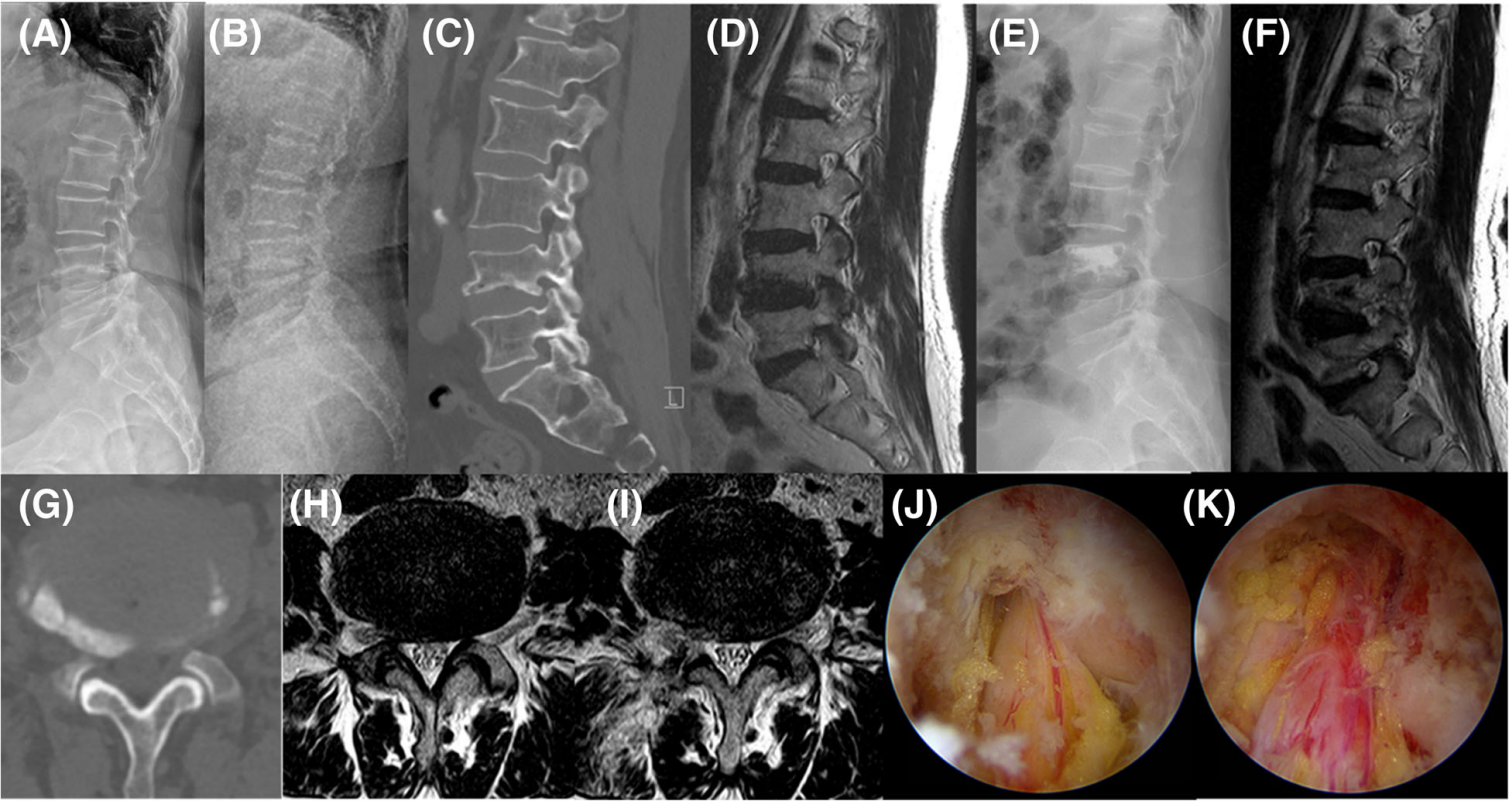
Acute L2 vertebral compression fracture sustained 2 months after surgery and treated with percutaneous VP. An 81-year-old female patient was diagnosed with L4 osteoporotic vertebral collapse (flat-type) and severe central canal and right central hugged lumbar disk herniation at L4-L5 underlying lumbar degenerative kyphosis. (**A**) Whole-spine lateral radiographic image (white dotted line: plum line). The patient simultaneously underwent (**B**) L4 percutaneous balloon kyphoplasty and (**C**) unilateral biportal endoscopic decompressive laminectomy. Sufficient neural decompression is seen on the magnetic resonance T2-weighted axial images (**D**) preoperatively, (**E**) immediately postoperatively, and (**F**) after 2 months postoperatively. Within the 2 months after surgery, she felt a significant improvement in pain and disability. Plain lateral radiographs (**G**) immediately postoperatively and (**H**) at 2 months postoperatively show acute L2 vertebral compression fracture, and (**I**) percutaneous vertebroplasty was performed on L2

As regards surgical complications, incidental durotomy was reported in 2 patients and epidural hematoma in another 2, but all patients improved with conservative treatment, and there was no need for re-operation. In one female patient, acute L2 vertebral compression fracture occurred when she fell 2 months after her surgery; however, she recovered without any complications after percutaneous VP was performed (Fig. 3).

## Discussion

The following were the major findings of this study: (1) posterior decompression assisted with a biportal endoscopic technique combined with anterior column stabilization by VP significantly reduced radicular pain and improved spinal disability in all patients; (2) the length of hospital stay for extremely elderly patients was quite short with less complication that could be improved by conservative treatment.

Recent reports have noted that osteoporotic fractures of the spine, hip, shoulder, and forearm lead to major health problems among the elderly population in developed countries [15]. In particular, some reports have suggested an increased risk of mortality in elderly people with osteoporotic fractures compared with the general population, regardless of age and sex [16]. Furthermore, the mortality risk is the highest in the first 5 years following all types of fractures. The major causes of death from death certificates were 27% cardiac, 26% respiratory, 15% cerebrovascular, and 13% malignancy. Fracture was mentioned in only 10.5% of death certificates, primarily hip and vertebral fracture, and osteoporosis without a fracture in an additional 2.5% [16]. While there is a controversy on whether early intervention for osteoporotic fractures may prevent some of these deaths, a study showed a decrease in mortality over time from the onset of a vertebral fracture and revealed that active intervention is required for early rehabilitation [17].

We compared the length of stay in this study with the existing literature on lumbar laminectomy and conservative treatment for vertebral compression fracture. According to a study by Yusuke Watanabe et al., conservative treatment for vertebral compression fractures over 65 years of age results in a longer hospital stay and an average hospital stay of 20.6 ± 4.4 ~ 40.0 ± 11.6 days [18]. In addition, according to a study by Takeshi Oichi et al., in a study comparing microendoscopic laminectomy and open laminectomy, the average length of hospital stay was 12 and 16 days, respectively. In old age, it is thought that the length of hospital stay is prolonged because there are many underlying medical conditions, and activities of daily living are separated [19]. In this respect, we think the average length of stay in this study was relatively short.

Delayed vertebral collapse in the thoracolumbar junction is often classified as a type 1 (wedge-type) vertebral fracture with severe kyphosis. Depending on which neural structure is affected, it can present various neurologic symptoms, including motor weakness, urinary problems, and/or sensory changes. On the contrary, neurologic complications are not frequently encountered in the lower lumbar region below L3, and the rate of surgical indication may be lower because the lower lumbar spine has a natural lordotic alignment and contains the cauda equina [2]. Nevertheless, flat or concave-type lower lumbar DVC may contribute to LSR or cauda equina syndrome by bone fragment retropulsion and traumatic segmental instability generating central canal and/or foraminal stenosis, all of which are known to occur frequently in extremely elderly patients [1, 2]. In this study, although the morphological features of DVC showed similar results to those of a previously published study, the clinical manifestations were unlike those in the previous study, which reported foraminal stenosis as the primary form of deterioration [2]. In this study, central canal stenosis was observed as the primary form of deterioration, presumably caused by traumatic lumbar disk herniation, vertebral height loss, traumatic segmental instability, and kyphosis. In this regard, we considered neural decompression of the central canals and the neural foramen for the recovery of the DVC-related LSR.

Some studies have reported an improvement in lower lumbar DVC-related LSR upon treatment with decompressive laminectomy and/or foraminotomy with posterior instrumented fusion [20]. However, there are concerns regarding surgical complications that may arise due to advanced age, patient frailty, and poor bone quality, which could eventually delay early rehabilitation and increase the risk of mortality. Therefore, some extremely elderly and high-risk patients require a new treatment strategy to minimize this risk and allow for early rehabilitation. In particular, VP, a classic minimally invasive form of treatment for OVCF that does not require general anesthesia, has reportedly shown relatively stable clinical outcomes in elderly patients [21]. Furthermore, the recently introduced BEPLD is a minimally invasive treatment modality that can produce sufficient neural decompression with minimal footprints and can lead to early recovery because of its low postoperative pain, reduced amount of estimated blood loss, minimal surgical site infections, and reversible paravertebral muscle damage [10, 22, 23]. Therefore, in this study, we applied BEPLD simultaneously with VP for the treatment of DVC-related neurologic deficit in extremely elderly patients, which resulted in a short hospital stay combined with stable clinical outcomes in the short-term follow-up period, without any significant perioperative or postoperative complications.

This study had certain limitations. First, this was a retrospective case review involving a small number of patients without a control group and has a short follow-up period. Second, DVC-related neurologic deficit is a relatively rare condition, and there were practical difficulties in the follow-up of the extremely elderly patients with reduced activity in the outpatient clinic for more than 6 months. We had to rely on electronic medical records or telephone interviews for the final follow-up data for these patients. Thus, the analysis of every parameter related to DVC was not possible. Finally, in this study, the therapeutic plan, including postoperative rehabilitation and possible complications, was fully explained to the patients and agreed upon before the surgery. Patients with degenerative global spinal sagittal imbalance were strongly advised on the need for anterior supported ambulation using a cane or walker during the postoperative rehabilitation. Even though VP can restore the vertebral height to a certain extent, there is a potential risk of the progression of a global spinal deformity, such as kyphosis in the long term and the consequent deterioration of this disability because BEPLD+VP aim to correct this accompanying deformity. Since this study focused solely on the clinical outcomes, the radiological assessment did not take into consideration the possibility of an accompanying degenerative coronal and/or sagittal spinal imbalance.

## Conclusions

BEPLD+VP is a type of salvage therapy that reduces surgical morbidity and the need for major spinal surgery under general anesthesia, while providing good clinical outcomes. Treatment of lower lumbar DVC-related LSR in extremely elderly patients is challenging; therefore, a new comparative clinical trial involving multiple institutions and long-term follow-up should be carried out in the future.

## Abbreviations

OVCFs: Osteoporotic vertebral compression fractures
DVC: Delayed vertebral collapse
LSR: Lumbosacral radiculopathy
BEPLD: Biportal endoscopic posterior lumbar decompression
VP: Vertebroplasty
WHO: World Health Organization
MRI: Magnetic resonance imaging
ASIA: American Spinal Injury Association
PMMA: Polymethyl methacrylate
VAS: Visual analog scale
mJOA: Modified Japanese Orthopedic Association
RR: Recovery rate
